# Emergency department diagnosis of supraspinatus tendon calcification and shoulder impingement syndrome using bedside ultrasonography

**DOI:** 10.1186/2036-7902-5-2

**Published:** 2013-02-11

**Authors:** David C Riley, Martha Kaufman, Theresa M Ward, Yesenia Acevedo, Rodney Guerra, Adenike Folorunsho

**Affiliations:** 1 Emergency Medicine Department, Columbia University Medical Center, New York, NY, USA

**Keywords:** Ultrasound, Emergency department, Supraspinatus tendon calcification, Impingement syndrome

## Abstract

A 45-year-old woman presented to the emergency department with a 2-day history of severe left shoulder pain made worse with movement. Emergency department (ED) bedside point-of-care static and dynamic ultrasound examination of the supraspinatus tendon revealed supraspinatus tendon calcification with impingement syndrome, and the patient was urgently referred to orthopedics after ED pain control was achieved. Bedside shoulder and supraspinatus tendon evaluation with static and dynamic ultrasonography can assist in the rapid diagnosis of supraspinatus tendon calcification and supraspinatus tendon impingement syndrome in the emergency department.

## Background

The prevalence of supraspinatus tendon calcification causing shoulder pain has been reported to be as high as 6.8%, mainly due to the supraspinatus tendon subacromial impingement syndrome with shoulder pain causing limited motion [1]. Radiology department diagnostic ultrasound evaluation of supraspinatus tendon calcification with subacromial impingement syndrome has been reported to be more specific (95% to 96%) than sensitive (71% to 81%) in adult patients, and supraspinatus tendon calcification with subacromial impingement syndrome diagnosed through ultrasonography has been reported in young athletes who perform overhead sports such as tennis, volleyball, and swimming [2-8]. Supraspinatus tendon calcification has also been reported to cause shoulder pain in children [9]. Undiagnosed and untreated supraspinatus tendon calcification with subacromial impingement syndrome can progress to severe forms of subacromial and subdeltoid bursitis, and even more serious surgical problems such as bicipital tendonitis with rupture of the long head of the biceps tendon, adhesive capsulitis, and rotator cuff tears [5-7]. Emergency physicians using point-of-care bedside ultrasonography to rapidly diagnose supraspinatus tendon calcification with subacromial impingement syndrome can expedite rapid musculoskeletal specialist referral to potentially improve patient outcomes.

## Case presentation

A 45-year-old right-handed woman with a past medical history of hypertension and stroke presented to the emergency department (ED) with a 2-day history of severe left shoulder progressively worsening pain and limited movement due to the pain. Aside from the pain on her left shoulder, the patient reported no associated trauma, weakness, numbness, left upper-extremity tingling, shortness of breath, chest pain, or fever. Her ED vital signs were as follows: temperature 36.9°C, blood pressure 132/70 mmHg, heart rate 73 bpm, respiratory rate 18 bpm, and oxygen saturation 98% on room air. The patient's electrocardiogram showed a normal sinus rhythm with no ST or T-wave abnormalities. Physical examination results were normal except for the left shoulder exam, where patient expressed severe pain with passive and active abduction of the humerus and some mild trapezial muscle and subacromial and subdeltoid area tenderness to palpation near the greater tuberosity of the humerus. The patient was able to actively abduct her left shoulder to 60°, but pain limited her ability to abduct further. The left arm was neurovascularly intact with normal motor and sensory functions of radial, median, ulnar, and axillary nerves. The patient had 5/5 motor strength of her shoulder upon internal and external rotation and with forearm supination; she stated no tenderness along her anterior shoulder near the long head of the biceps tendon in the bicipital groove.

**Figure 1:**
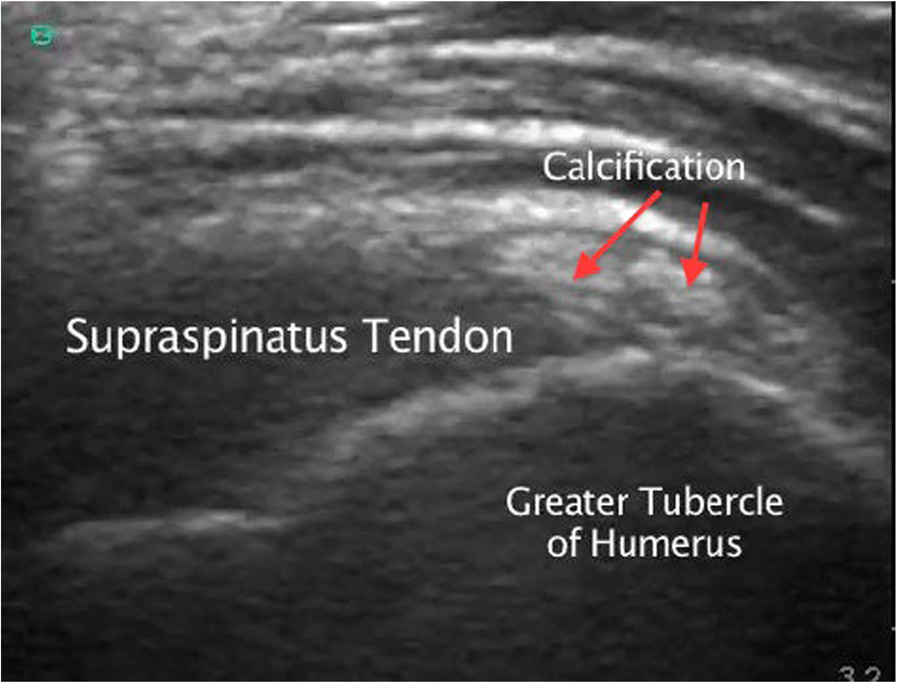
Supraspinatus tendon calcification.

**Figure 2:**
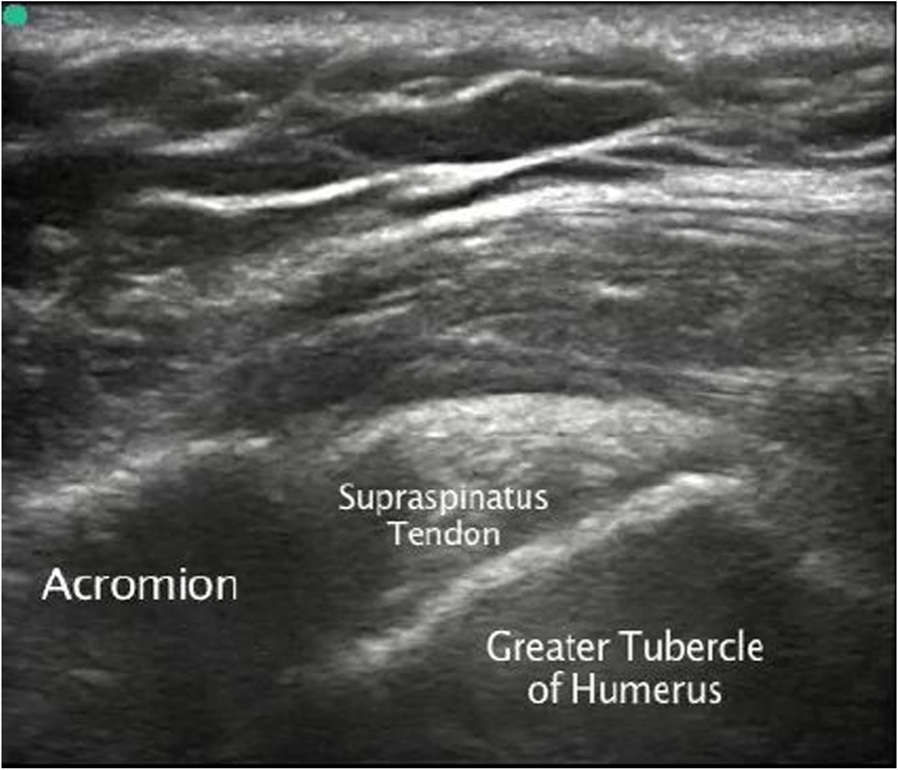
Supraspinatus tendon impingement under the acromion.

On initial assessment, the triage nurse suspecting possible shoulder calcific tendonitis, considering the severe pain that the patient was experiencing, notified the ED physician assistant and the ED physician to facilitate rapid pain medication administration and a rapid bedside ED ultrasound evaluation of the patient's left shoulder. A focused bedside ED ultrasound examination of the patient's supraspinatus tendon was performed by an ultrasonographer ED attending physician, with more than 10 years of experience, after administering opiate analgesia to the patient (see Additional files 1 and 2 available as supporting information in the online version of this paper). With the patient in modified Crass position (where the patient placed her palm on her ipsilateral iliac wing and moved her elbow as posterior as possible), long-axis evaluation of the patient's supraspinatus tendon revealed calcification near the attachment to the greater tuberosity of the humerus, and a dynamic bedside ultrasound long-axis evaluation of the patient's supraspinatus tendon showed elevation of the greater tuberosity cranially to the level of the acromion and impingement of the supraspinatus tendon underneath the acromion of the scapula when the patient actively abducted her left humerus (Figures 1 and 2; Additional files 1 and 2). With a dynamic humerus abduction maneuver, we were able to directly see the calcified and thickened supraspinatus tendon causing the impingement syndrome with limited abduction, and this would not be possible with X-ray calcification diagnosis alone.

**Figure 3:**
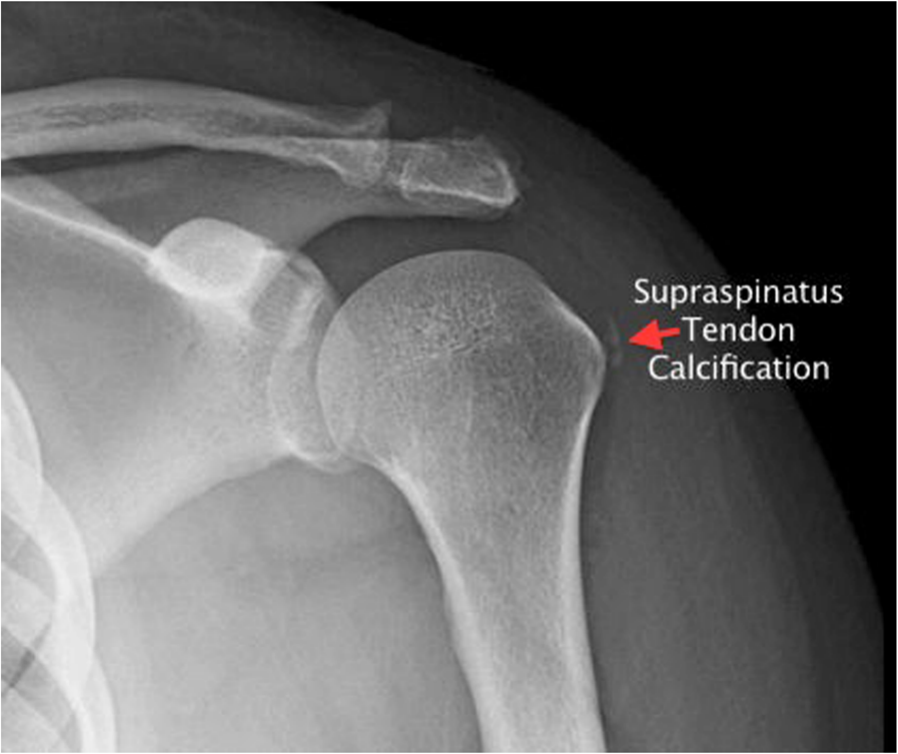
Plain film radiograph of the proximal humerus with supraspinatus tendon calcification.

In the radiology department, proximal humerus plain film radiography showed two small soft tissue calcifications in the region of the supraspinatus tendon of the left shoulder, likely thought to be calcific tendinitis (Figure 3). The patient's pain was controlled in the ED, and she was treated and released with oral pain medications, a left arm sling, and urgent orthopedic follow-up.

## Discussion

Supraspinatus tendon calcification is thought to be due to the deposition of calcium hydroxyapatite crystals inside the supraspinatus tendon near the greater tuberosity of the humerus insertion point, and the calcium deposits in the supraspinatus tendon may be due to fibrosis, necrosis, tendon degeneration, or systemic non-degenerative causes [1,8]. Supraspinatus tendon calcification may lead to tendon thickening which could lead to impingement under the acromion of the scapula, causing an impingement syndrome and severe pain upon humerus abduction [1].

Dr. Charles Neer in 1972 was the first person to describe subacromial impingement syndrome as a distinct clinical problem [2,3,7]. Neer described three stages of subacromial impingement: stage I impingement involves edema and hemorrhage of the subacromial-subdeltoid bursa and rotator cuff, and typically occurs in patients less than 25 years old; stage II impingement involves tendinopathy, such as fibrosis or tendinosis and calcific tendonitis, usually occurring in patients 25 to 40 years old; and stage III impingement typically involves surgical problems such as a rotator cuff tear and usually manifests in patients older than 40 years old [3,7]. Bigliani and Levine have classified the causes of subacromial impingement syndrome as either intrinsic/intratendinous or extrinsic/extratendinous, and each group can be either a primary etiology that directly causes the impingement or a secondary etiology which is the result of another process such as instability or neurologic injury [7]. Examples of intrinsic primary impingement are degenerative tendinopathy and calcification of the supraspinatus tendon that can lead to tendon thickening and impingement, the most likely etiology of our patient's supraspinatus tendon subacromial impingement and shoulder pain. Examples of extrinsic primary impingement include thickening of the coracoacromial ligament and a hook-shaped acromial scapular bone because a hook-shaped acromial bone is more likely to be present in patients with subacromial impingement syndrome and with patients with rotator cuff tears compared to patients who have a flat- or curve-shaped acromial bone [7]. Emergency physicians should consider the diagnosis of supraspinatus tendon calcification with subacromial impingement syndrome in patients with shoulder pain to expedite urgent musculoskeletal specialist referral.

Radiologists have developed a full five-step ultrasonographic shoulder protocol that includes evaluation of the following structures: the long head of the biceps brachii tendon, the subscapularis tendon, the supraspinatus tendon and rotator interval with both static and dynamic evaluation for subacromial impingement, the acromioclavicular joint, and the infraspinatus and teres minor tendons with the posterior glenoid labrum [10]. The performed ultrasonography is 79% sensitive and 88% specific for diagnosing supraspinatus tendon calcification and impingement syndrome using dynamic maneuvers such as humerus abduction [11]. One of the key pitfalls for all musculoskeletal ultrasound examinations is anisotropy artifact that requires perpendicular insonation of tendons, ligaments, muscle, and nerves to observe the correct echotexture of the structures [10]. Another pitfall is the improper positioning of the shoulder in visualizing the supraspinatus tendon. Radiologists have also developed specific sonographic signs of shoulder subacromial impingement such as pooling of fluid laterally to the subdeltoid portion of the subacromial-subdeltoid bursa while the humerus is abducted [5]. Our patient had excellent internal and external rotation motor strength of her shoulder, and she stated no tenderness over her long head of the biceps tendon. A focused point-of-care emergency department bedside ultrasound examination was performed over her supraspinatus tendon, and this revealed calcification near the attachment to the greater tuberosity of the humerus; a dynamic bedside ultrasound long-axis evaluation of the patient's supraspinatus tendon showed elevation of the greater tuberosity cranially to the level of the acromion and impingement of the supraspinatus tendon underneath the acromion of the scapula when the patient actively abducted her left humerus. A complete evaluation of our patient's supraspinatus tendon was obstructed by the calcifications in her supraspinatus tendon, and which may have hidden a partial rotator cuff tear.

Musculoskeletal ultrasonography is an operator-dependent imaging modality, yet the inter-rater reliability of shoulder ultrasonography performed in the radiology department to detect supraspinatus tendon calcification among inexperienced radiologists (6 months of experience) versus experienced radiologists (6 years of experience) has been reported as kappa of 0.70 to 0.83, substantial agreement [12]. No reliability data are currently available for emergency physicians in performing shoulder ultrasonographic examinations.

The initial treatment for supraspinatus tendon calcification with subacromial impingement syndrome is pain control and rest, as some patients will improve with conservative therapy [1]. Many additional treatment modalities for supraspinatus tendon calcification with subacromial impingement syndrome are available including extracorporeal shock wave therapy, diathermy hyperthermia therapy, ultrasound-guided percutaneous needle aspiration and lavage, and more invasive arthroscopic and open orthopedic surgical procedures [13-20]. Patients with supraspinatus tendon calcification with subacromial impingement syndrome, who undergo ultrasound-guided percutaneous needle aspiration and lavage with corticosteroid injections into the subdeltoid-subacromial bursa have been found to have prompt shoulder function recovery after the procedure and better outcomes at 1-year follow-up; however, at five and ten years, the non-needle aspirated and lavage group reported outcomes similar to the needle aspirated and lavage group [20]. Emergency department primary therapy will include oral pain medication, rest, provision of an arm sling, and urgent musculoskeletal specialist referral. Emergency department ultrasonography can help in preventing missed or delayed diagnosis of supraspinatus tendon calcification with subacromial impingement syndrome when the diagnosis is not always clear clinically, and bedside point-of-care ultrasound is of great utility in cases where physical examination maneuvers can be limited by pain and soft tissue swelling.

## Conclusion

Emergency-physician performed bedside point-of-care shoulder musculoskeletal ultrasonography can assist in the rapid diagnosis of supraspinatus tendon calcification and impingement syndrome in the emergency department. Rapid diagnosis of supraspinatus tendon calcification and impingement syndrome can expedite shoulder musculoskeletal consultation and treatment.

## Consent

Written informed consent was obtained from the patient for publication of this Case Report and any accompanying images. A copy of the written consent is available for review by the Editor-in-Chief of this journal.

## Competing interests

The authors declare that they have no competing interests.

## Authors’ contributions

DR, MK, TW, YA, RG, and AF drafted and edited the manuscript. All authors read and approved the final manuscript.

## Authors’ information

DR is the Director of Emergency Ultrasonography and Ultrasound Research. MK and YA are emergency department nurses. TW and RG are physician assistants at the Emergency Medicine Department, Columbia University Medical Center, New York. AF is an emergency medicine resident in the New York Presbyterian, Columbia/Cornell training program, New York.

## Supplementary Material

Additional file 1 **Emergency Department ultrasonography bedside diagnosis of supraspinatus tendon calcification.** Video of long-axis evaluation of the supraspinatus tendon calcification near the humerus greater tuberosity attachment. Click here for file

Additional file 2 **Emergency Department ultrasonography bedside diagnosis of supraspinatus tendon impingement syndrome.** Video of long-axis evaluation of the supraspinatus tendon impingement underneath the acromion of the scapula. Click here for file
